# Second- and third-line treatment strategies in multiple myeloma: a referral-center experience

**DOI:** 10.1038/s41408-022-00757-8

**Published:** 2022-12-06

**Authors:** Sarah Goldman-Mazur, Alissa Visram, S. Vincent Rajkumar, Prashant Kapoor, Angela Dispenzieri, Martha Q. Lacy, Morie A. Gertz, Francis K. Buadi, Suzanne R. Hayman, David Dingli, Taxiarchis Kourelis, Wilson Gonsalves, Rahma Warsame, Eli Muchtar, Nelson Leung, Robert A. Kyle, Shaji K. Kumar

**Affiliations:** 1 Division of Hematology, Mayo Clinic Rochester, Rochester, MN USA; 2 Division of Hematology, Ottawa Hospital Research Institute, Ottawa, ON Canada; 3 Division of Nephrology, Mayo Clinic Rochester, Rochester, MN USA

**Keywords:** Myeloma, Epidemiology

## Abstract

The treatment landscape for relapsed multiple myeloma (MM) has increased. In this study, we aimed to characterize 2nd (*n* = 1439) and 3rd (*n* = 1104) line regimens and compare the results between subgroups based on the year of treatment initiation (2nd line: 2003–2008, 2009–2015, 2016–2021; 3rd line: 2004–2009, 2010–2015, and 2016–2021). In both the second- and third- lines, we observed increasing use of novel agents (from 78 to 95% and from 77 to 95%, respectively) and triplet regimens (from 15 to 69% and from 21 to 71%, respectively). The most frequently used regimens in the last studied periods included lenalidomide-dexamethasone (RD; 14%), carfilzomib-RD (12%), and daratumumab-RD (10%) for the second-line, and daratumumab-pomalidomide-dexamethasone (11%) and daratumumab-RD (10%) for the third-line. The median time to the next treatment from second-line therapy has improved from 10.4 months (95% CI: 8.4–12.4) to 16.6 months (95% CI: 13.3–20.3; *p* < 0.001). The median overall survival from the first relapse increased from 30.9 months (95% CI: 26.8–183.0) to 65.8 months (95% CI: 50.7–72.8; *p* < 0.001). Over the last two decades, more patients were treated with newer agents and triplets for relapsed MM. The landscape of regimens has become more diverse, and survival after the first relapse is continually improving.

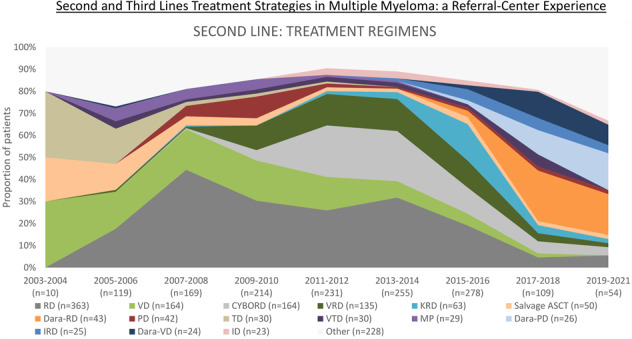

## Introduction

The choice of second-line therapy in multiple myeloma (MM) is based on several factors, including agents used in the first-line, response to prior treatment, the aggressiveness of the relapse, refractoriness to lenalidomide, and performance status [1]. Over the past decades, the landscape of second-line therapies was shaped by the introduction of novel agents, favorable results from clinical trials, different side-effects profiles, and attempts at a more individualized treatment approach. The recommendations for second-line treatment started with high-dose dexamethasone, salvage autologous stem cell transplantation (ASCT), and thalidomide-based therapy before 2003 [2], through treatments based on lenalidomide, bortezomib, or alkylators [3] and current recommendations for daratumumab (or other novel agents, including carfilzomib, pomalidomide, elotuzumab, or isatuximab)-based therapy [1]. Despite these immense changes and growing diversity of second-line therapies, the treatment landscape for relapsed MM outside of clinical trials and the effectiveness of various regimens has not been studied systematically.

Recently, Jagannath et al. described the heterogeneity of second-line therapies based on data from the mostly community-based registry from the United States, *Connect MM*, which encompasses second-line therapies implemented between 2010 and 2016 [4]. They concluded that treatment choices coincided with the approval status of newer agents, and community physicians are up to date with the newest recommendations. Over the studied 6-year period, triplet combinations and newer agents (carfilzomib, pomalidomide, daratumumab, and elotuzumab) started to be used more frequently.

We designed this study to characterize how second-line treatment strategies have evolved over the last two decades for MM patients based on data from our institutional database. Next, we aimed to show if the effectiveness of second-line therapies (reflected by post-progression survivals) has improved over time and the impact of specific regimens. Finally, among patients who progressed to a subsequent line of therapy, we looked at the treatment regimens used in the third-line.

## Methods

### Case selection

We retrospectively assessed relapsed MM patients seen at Mayo Clinic, Rochester, diagnosed between February 2001, and December 2018. The cohort included patients 18 years or older, diagnosed with MM with at least one disease relapse that required an additional line of treatment.

We collected clinical and laboratory data at diagnosis, data on treatment regimens, progression, and overall survival (OS). Time to next treatment (TTNT) was defined as the time between initiation of second- and third-line therapy, and this variable was available for 1239 patients (for 103, the details of the second progression were unknown, for the remaining 97, the date of starting third-line treatment was not available). Patients who have not progressed to a subsequent line of therapy were censored at the date of the last follow-up or at death. High-risk fluorescence in situ hybridization has been defined as the presence of deletion 17p/TP53 mutation and/or translocation t(4;14), t(14;16), or t(14;20) [5]. Immunomodulatory drugs (IMIDs) include thalidomide, lenalidomide, and pomalidomide; proteasome inhibitors (PIs) include bortezomib, carfilzomib, and ixazomib. Novel agents were defined as lenalidomide and next-generation IMIDs (excluding thalidomide), bortezomib, and next-generation PIs, and daratumumab. All patients authorized the use of their medical record data for research [6]. The study was approved by the Mayo Clinic Institutional Review Board.

### Statistical analysis

To visualize trends in treatment choices, we divided the study period into nine 2-year intervals, and for descriptive purposes, the period was divided into three 6-year intervals. We used “100% stacked area” charts to show how the constituent parts of the whole have changed over time. The height of each colored stack represents the proportion of patients in that category at a given point in time.

Data are expressed as mean and standard deviation or median (interquartile range, IQR) as appropriate. The Shapiro–Wilk test was used to assess conformity with a normal distribution. The continuous variables were compared between two groups using the Student’s *t*-test for independent groups for mean values and Mann–Whitney *U* test for distribution. Categorical variables were analyzed using the *χ*^2^ test or Fisher’s exact test as appropriate. For the survival analysis, the Kaplan–Meier method was used to generate survival curves, which were then compared using the log-rank test. Statistical analysis was performed with STATISTICA 12.0 (StatSoft, Tulsa, OK).

## Results

### Baseline patient characteristics and first-line therapy

A total of 1439 patients were included in the study, and the baseline characteristics are presented in Supplementary Table 1. Patients were diagnosed between 2001 and 2018, and the initiation of second-line treatment occurred between June 2003 and February 2021. The median age at diagnosis was 62.7 years, and 60.1% were male. No significant differences between subgroups divided by the year of relapse (2003–2008, 2009–2014, and 2015–2021) were observed, except for the higher prevalence of extramedullary plasmacytomas at diagnosis and higher serum beta-2-microglobulin levels in patients identified with relapse in the most recent period between 2015 and 2021.

In the first-line therapy, novel agents were used in 1319 cases (91.7%, Supplementary Fig. 1); regimens based on PIs in 26.5%, IMIDs in 42.0%, and a combination of PI + IMID in 21.2% of patients. Upfront single ASCT was performed in 689 patients (47.9%), tandem ASCT in 29 patients (2.0%), and allogeneic stem cell transplantation (allo-SCT) in 3 patients (0.2%). Maintenance after first-line therapy was used in 362 patients (25.2%), including 194 cases (13.5%) of lenalidomide-based maintenance and 125 cases (8.7%) of bortezomib-based maintenance.

### Second-line therapy and changes over time

**Fig. 1:**
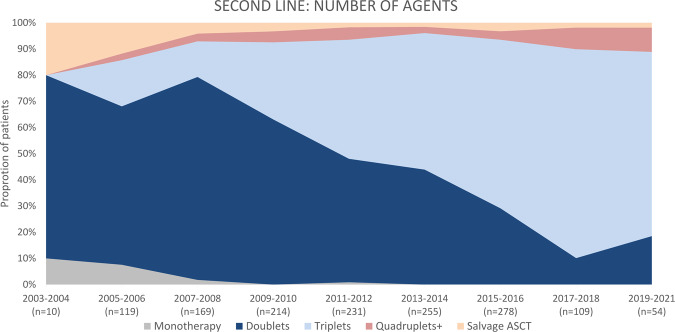
Number of agents applied as second-line treatment in multiple myeloma patients, initiation of second-line treatment 2003–2021. ASCT autologous stem cell transplantation.

**Fig. 2:**
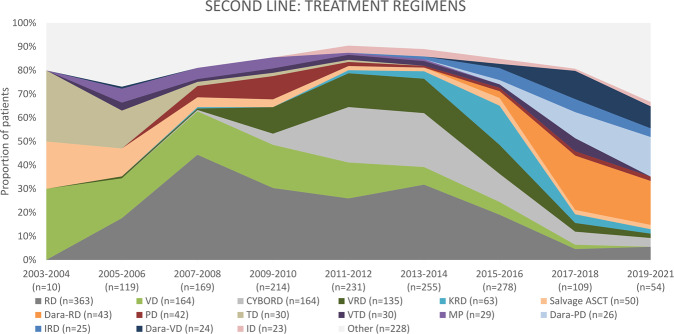
Most frequently applied second-line treatment regimens in multiple myeloma patients, initiation of second-line treatment 2003–2021. CYBORD cyclophosphamide-bortezomib-dexamethasone, D dexamethasone, Dara daratumumab, I ixazomib, K carfilzomib, MP melphalan-prednisone, P pomalidomide, R lenalidomide, T thalidomide, V bortezomib.

The years 2003–2008 include 298 patients. For second-line treatment, during 2003–2008, 233 patients (78.2%) received novel agents. The majority were treated with doublets (*n* = 210, 70.5%), followed by triplets (*n* = 44, 14.8%) and salvage ASCT (*n* = 23, 7.7%) (Fig. 1). The most frequently used regimens were lenalidomide-dexamethasone (RD, *n* = 96, 32.2%), bortezomib-dexamethasone (VD, *n* = 55, 18.5%), followed by thalidomide-dexamethasone (TD, *n* = 25, 8.4%) and salvage ASCT (*n* = 24, 8.1%) (Fig. 2). The rest of the implemented regimens is listed in Supplementary Table 2. In addition to salvage ASCT, 31 patients (10.4%) underwent single consolidative ASCT (after achieving the second remission), 1 (0.3%) tandem ASCT, and 3 patients (1.0%) underwent allo-SCT. Sixteen patients (5.4%) were treated with maintenance/consolidation, including six patients with lenalidomide- and two with bortezomib-based treatments.

Between 2009 and 2014, 700 patients were treated for relapse, and 648 (92.6%) received novel agents. The use of triplets in the second-line increased (*n* = 301, 43.0%), although doublets were still more common (*n* = 356, 50.9%). Most frequently used regimens included: RD (*n* = 206, 29.4%), bortezomib-cyclophosphamide-dexamethasone (CYBORD, *n* = 122, 17.4%), bortezomib-lenalidomide-dexamethasone (*n* = 94, VRD, 13.4%) and VD (*n* = 93, 13.3%). After achieving response to second-line treatment, 110 patients (15.7%) underwent consolidative ASCT and 7 patients (1.0%) allo-SCT; 93 (13.3%) received maintenance/consolidation, including 31 lenalidomide- and 43 bortezomib-based treatments.

Finally, during 2015–2021, 441 were included, and 425 (96.4%) received novel agents. Triplets were most common (*n* = 304, 68.9%), followed by doublets (*n* = 102, 23.0%). Most frequently used regimens include RD (*n* = 61, 13.8%), carfilzomib-RD (*n* = 52, 11.8%), daratumumab-RD (Dara-RD; *n* = 45, 10.2%), CYBORD (*n* = 41, 9.3%), VRD (*n* = 40, 9.1%), Dara-pomalidomide-dexamethasone (Dara-PD; *n* = 26, 5.9%) and Dara-VD (*n* = 23, 5.2%). Fifty-eight patients (13.2%) underwent single consolidative ASCT after second-line treatment, 1 (0.2%) underwent tandem ASCT; maintenance/consolidation was applied in 92 patients (20.9%), including 41 (9.3%) lenalidomide- and 16 (3.6%) bortezomib-based treatments.

### Progression on treatment/maintenance- impact on second-line treatment choices

Progression on treatment (either active or maintenance) occurred in 601 patients (41.8%) and influenced the choice of second-line therapy in comparison to patients relapsing while off treatment (*n* = 838, 58.2%). This includes more frequent use of quadruplets in comparison to patients who relapsed being off treatment (*n* = 42, 6.9% vs. *n* = 15, 1.8%; *p* < 0.001), the use of salvage ASCT (*n* = 32, 5.3% vs. *n* = 19, 2.3%; *p* = 0.002), treatment based on PIs (*n* = 352, 58.6% vs. *n* = 365, 43.6%; *p* < 0.001) or PI + IMID (*n* = 145, 24.1% vs. *n* = 128, 15.3%; *p* < 0.001) and the use of monoclonal antibodies (*n* = 80, 13.3% vs. *n* = 45, 5.4%; *p* < 0.001). Patients who relapsed on active treatment/maintenance were treated less often with an IMID-based regimen (*n* = 290, 48.3% vs. *n* = 546, 65.2%; *p* < 0.001).

One hundred twenty-three patients relapsed on lenalidomide maintenance. The most commonly used second-line strategies in those patients were CYBORD (*n* = 20, 16.3%), VD (*n* = 14, 11.4%), Dara-VD (*n* = 12, 9.8%), followed by Dara-PD (*n* = 9, 7.3%).

### Survival over two decades

**Fig. 3:**
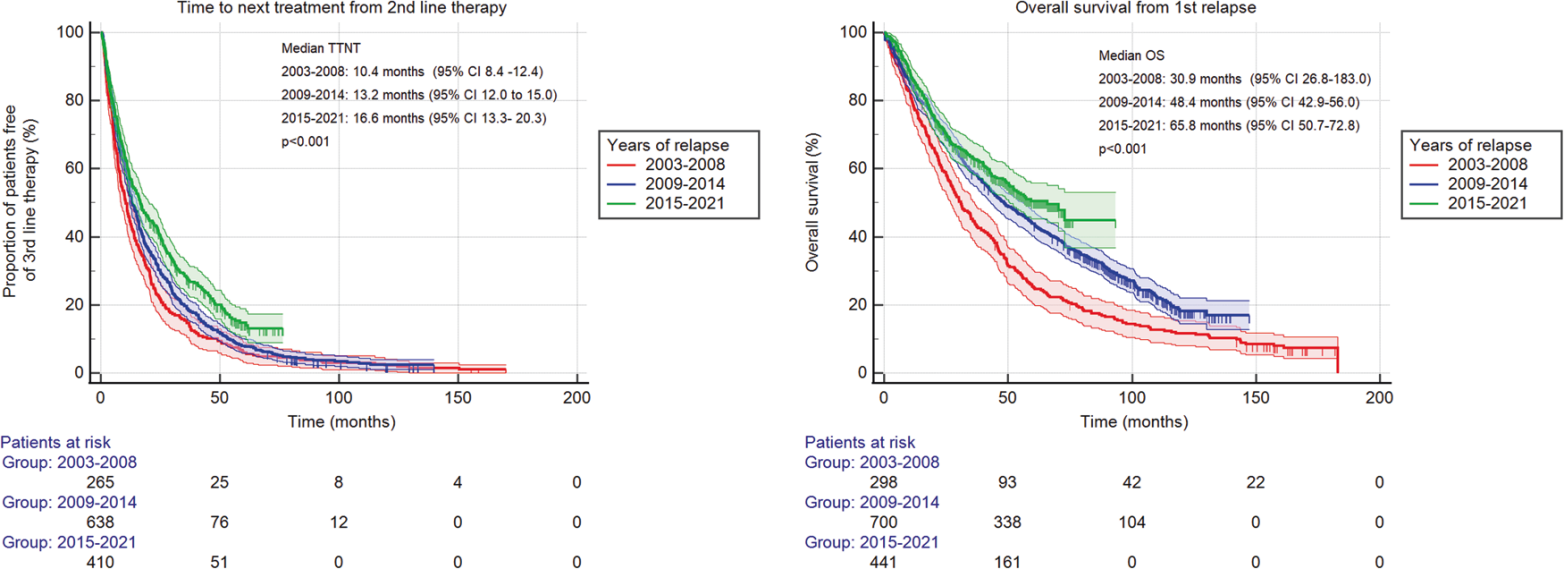
Time to next treatment (TTNT) and overall survival (OS) from first relapse estimates in 1439 treated patients with multiple myeloma stratified by the year of initiation of second-line therapy. CI confidence interval.

Median TTNT from second-line therapy has improved over the time of the study (*p* < 0.01; Fig. 3). Similarly, the median OS from the first relapse has increased over the three-time intervals.

### Impact of second-line regimens on survival

**Fig. 4:**
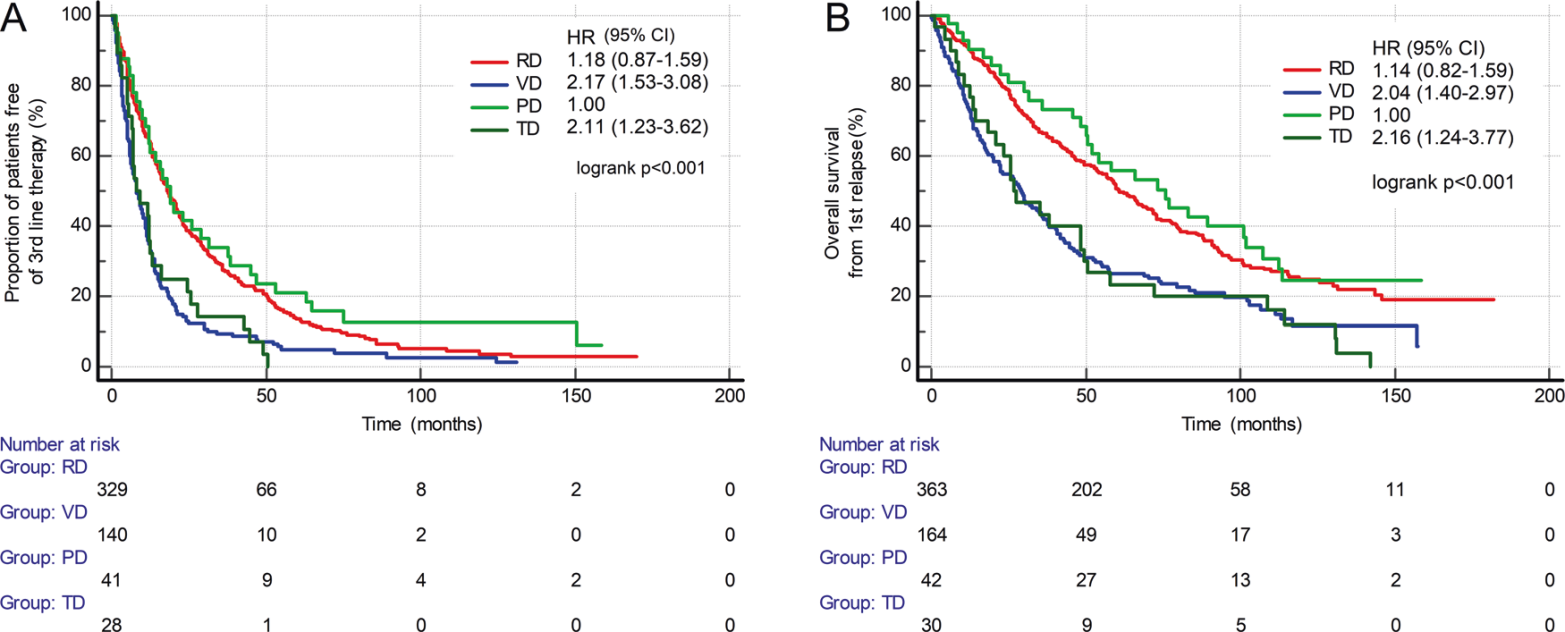
Comparisons of most frequently used doublet regimens on post-progression survival. **A** Time to next treatment from first relapse (TTNT). **B** Overall survival from first relapse (OS). CI confidential interval, HR hazard ratio, PD pomalidomide-dexamethasone, RD lenalidomide-dexamethasone, TD thalidomide-dexamethasone, VD bortezomib-dexamethasone.

Among the most frequently used doublet regimens (RD vs. VD vs. PD vs. TD), the longest survivals were observed in patients who received RD, and PD, including both TTNT from second-line therapy and OS from first relapse (Fig. 4). For the PD and RD group, the median TTNT from second-line therapy was 18.2 months (95% CI: 15.6–21.1) and 19.0 months (95% CI 12.0–31.6), respectively (Fig. 4A). Median OS from the first relapse was 60.7 months (95% CI: 54.1–71.3) in the PD group and 75.7 months (95% CI: 50.0–101.8) in the RD group (Fig. 4B).

**Fig. 5:**
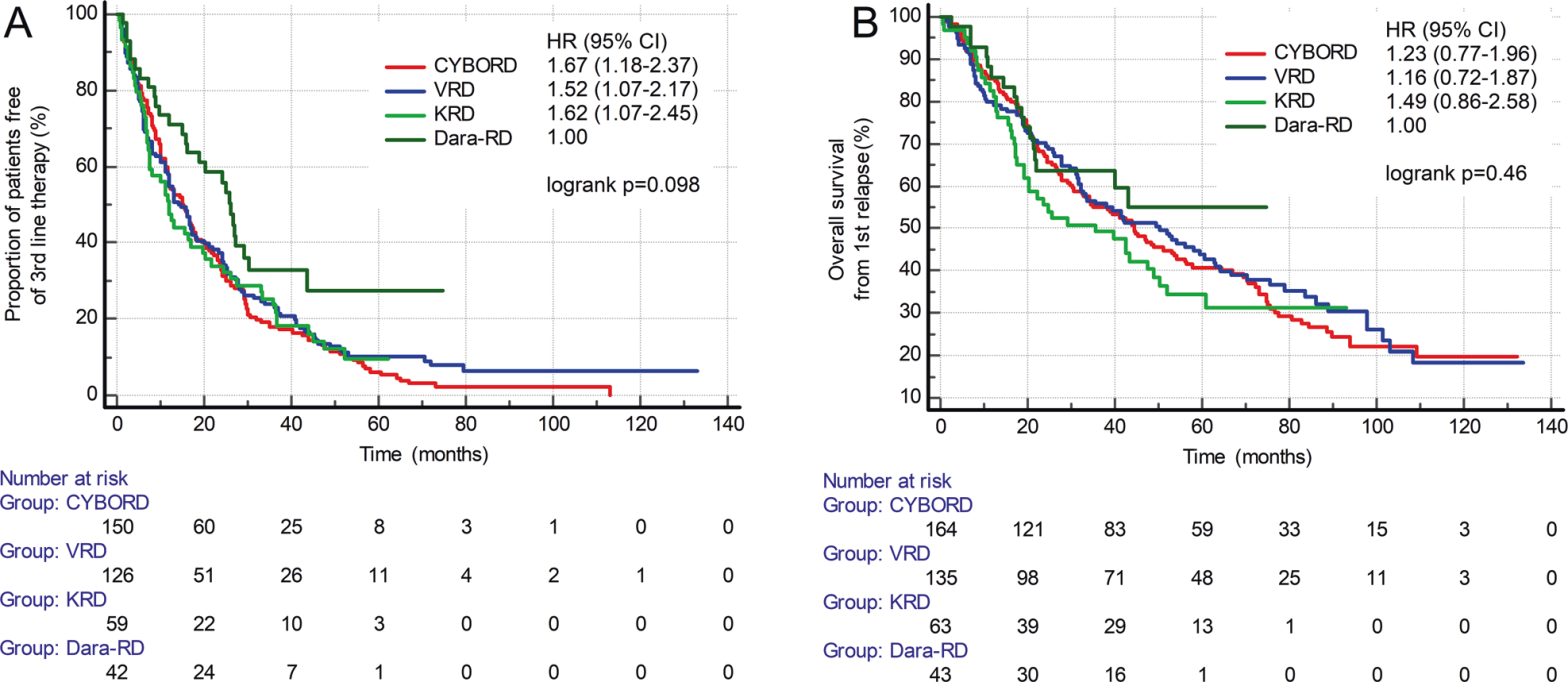
Comparisons of most frequently used triplet regimens on post-progression survival. **A** Time to next treatment from first relapse (TTNT). **B** Overall survival from first relapse (OS). CI confidence interval, CYBORD cyclophosphamide-bortezomib-dexamethasone, Dara-RD daratumumab-lenaliodmide-dexamethasone, HR hazard ratio, KRD carfilzomib-lenalidomide-dexamethasone, VRD bortezomib-lenalidomide-dexamethasone.

Among the most common triplet regimens (CYBORD vs. VRD vs. carfilzomib-RD vs. Dara-RD), the longest survivals were observed in patients who received Dara-RD (Fig. 5), although the difference was not statistically significant. In the Dara-RD group, the median TTNT from second-line therapy was 26.0 months (95% CI: 15.7–30.3, Fig. 5A), and the median OS from the first relapse was not reached (Fig. 5B).

### Third-line therapy and its changes over time

Of 1439 patients, 1173 had a second relapse, and 163 had not progressed at the time of follow-up. For 103, the status of progression was unknown. Third-line therapy was known for 1133 patients, 29 received hospice/supportive care, and in 1104 third-line therapy was implemented, the treatment started between August 2004 and April 2021. Like second-line therapy, the number of agents used and the variety of regimens in the third-line started to increase.

**Fig. 6:**
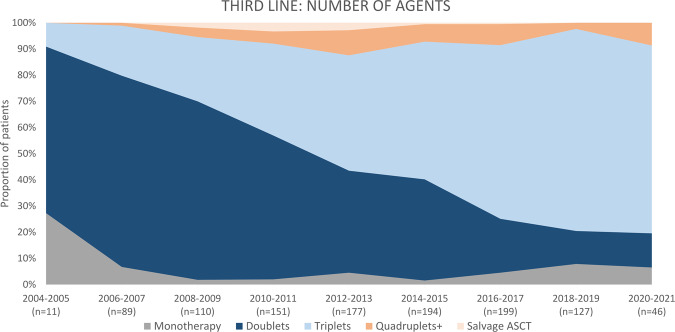
Number of agents applied as third-line treatment in multiple myeloma patients, initiation of third-line treatment 2004–2021. ASCT autologous stem cell transplantation.

**Fig. 7:**
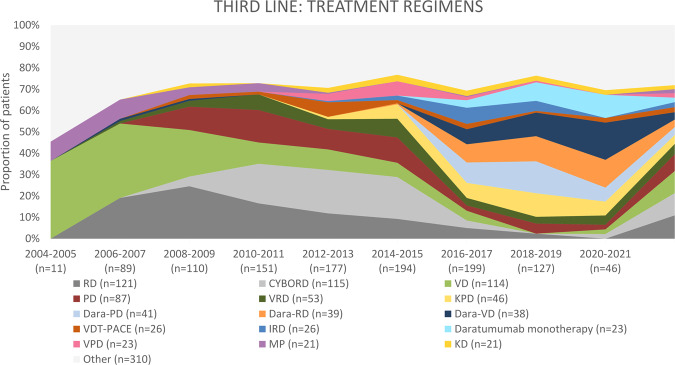
Most frequently applied third-line treatment regimens in multiple myeloma patients, initiation of third-line treatment 2004–2021. CYBORD cyclophosphamide-bortezomib-dexamethasone, D dexamethasone, Dara daratumumab, I ixazomib, K carfilzomib, MP melphalan-prednisone, P pomalidomide, R lenalidomide, T thalidomide, V bortezomib, VDT-PACE bortezomib, dexamethasone, thalidomide, cisplatin, doxorubicin, cyclophosphamide, and etoposide.

Between 2004 and 2009, 210 patients were treated for the second relapse, and 161 patients (76.7%) received novel agents. The majority (*n* = 147, 70.0%) was treated with doublets, followed by triplets (*n* = 45, 21.4%; Fig. 6). The most frequently used regimens were VD (*n* = 59, 28.1%), RD (*n* = 44, 20.1%), melphalan-prednisone (MP; *n* = 13, 6.2%), and PD (*n* = 12, 5.7%; Fig. 7). ASCT was used in six patients (2.9%), including two salvage ASCT (1.0%). Four patients received allo-SCT (1.9%). Two patients (1.0%) received bortezomib-based maintenance.

Next, 522 patients received third-line therapy between 2010 and 2015. Almost 89% (*n* = 464) received novel agents. Most patients received triplet regimens (*n* = 233, 44.6%), followed by doublets in 227 patients (43.5%) and quadruplets+ (*n* = 37, 7.1%). The most frequently used regimens included: CYBORD (*n* = 102, 19.5%), RD (*n* = 64, 12.3%), PD (*n* = 63, 12.1%), and VD (*n* = 45, 8.6%). ASCT was implemented in 29 patients (5.6%), including 11 patients who received salvage ASCT (2.1%). Two patients (0.4%) received pomalidomide-based maintenance and two bortezomib-based maintenance.

Finally, between 2016 and 2021, 372 patients were treated due to a second relapse. Over 95% of patients received novel agents (*n* = 359). The majority received triplet regimens (*n* = 263, 70.7%), and only 63 patients (16.9%) received doublet regimens. Dara-PD was the most frequently used regimen (*n* = 41, 11.0%), followed by Dara-RD (*n* = 38, 10.2%), Dara-VD (*n* = 36, 9.7%), and carfilzomib-PD (*n* = 31, 8.3%). Eight patients (2.2%) received ASCT, including 1 patient (0.3%) with salvage ASCT. One patient (0.3%) received allo-SCT. Three patients (0.8%) received maintenance (2 Dara-, 1 bortezomib-based).

## Discussion

Over the past two decades, increasing numbers of patients were treated with novel agents for the first relapse, reaching over 96% in the last 6 years and triplets started to be more popular. Although IMID-based therapies dominated, with RD being the most frequent regimen throughout the whole studied period, the role of combined PI + IMID-based therapies started to increase (reflected first by CYBORD and VRD and in the last studied period by regimens using carfilzomib and daratumumab), along with the use of monoclonal antibodies. The variety of regimens used has also notably increased. During the studied period, the use of consolidative ASCT in the second-line remained similar (10–15%), although the use of maintenance after the second-line started to increase (from 5% up to 20%). In the study by Jagannath et al., similar trends have been described [4]. The study comprised 855 patients who received second-line therapy between 2010 and 2016. The most used regimen included therapies based on lenalidomide and/or bortezomib, and the number of patients receiving triplet increased throughout the studied period. The authors also observed the increasing role of novel agents, such as carfilzomib, pomalidomide, daratumumab, and elotuzumab, over the years. This is in line with our results from the corresponding period (2009–2014) when RD, CYBORD, and VRD were most frequently used. The changes in third-line therapy were similar to the one described for second-line: the number of patients treated with novel agents and triplets increased, and the role of combined PI + IMID-based regimens also started to increase, with regimens based on daratumumab, pomalidomide, lenalidomide, and carfilzomib being the most popular in the past 6 years. The use of ASCT in the third-line, however, was minimal (approximately 3% of patients), so the use of maintenance after the third-line.

Our study shows continued improvement of survival after the first relapse, confirmed in both OS from second-line therapy and TTNT from the first relapse, which more directly reflects the role of the used second-line regimen. The general improvement in the survival of MM patients has already been observed and confirmed by our group, and the survival benefit was linked to the use of novel agents in first-line therapy [7]. Randomized trials have already shown the efficacy of new agents given alone or with dexamethasone in relapsed MM [8–12]. The addition of a new agent to a backbone two-drug combination (triplets vs doublets) was also superior to standard therapies [13–16]. Therefore, we suggest that the improvement in survival seen in our study is mostly driven by the increasing use of newer agents and triplets in relapsed MM patients.

The increasing number of available combinations of drugs in clinical practice enables further individualized approaches in treatment with a better balance of benefits and complications. On the other hand, it also constitutes a growing problem in adequate comparisons of effectiveness [17]. The recommended regimens for first relapse are based mainly on the information on whether the patient is lenalidomide and/or bortezomib sensitive, and the list of available options is long, and meeting the perfect goal can be more challenging with the growing possibilities [18, 19]. The head-to-head comparisons of different regimens presented in our study need to be interpreted with great caution since the settings were not standardized, the patient’s previous treatment was not analyzed, and the analysis was retrospective. Nonetheless, looking at the curves, we can hypothesize that therapy based on new drugs- lenalidomide, pomalidomide, and daratumumab- possess the potential for improving survival and should be used whenever possible.

In conclusion, over the past two decades, the effectiveness of second-line treatment has improved. Triplet therapies started to be used more frequently, both in the second- and third-lines of treatment, and the landscape of treatment regimens for relapsed MM patients has become more diverse, which may reflect a more individualized approach to each patient. However, the large variety of treatment strategies makes comparisons more and more challenging.

## Supplementary information

Supplementary Tables and Figures in PDF

## Data Availability

The datasets generated during and analyzed during the current study are available from the corresponding author upon reasonable request.
