# Supplementary material for: Second- and third-line treatment strategies in multiple myeloma: a referral-center experience
Source: Blood Cancer J. 2022 Dec 6;12(12):164. doi: 10.1038/s41408-022-00757-8 (PMC9723783; doi:10.1038/s41408-022-00757-8)
Supplement: Supplementary file 1 — Supplementary Tables and Figures in PDF [file 41408_2022_757_MOESM1_ESM.pdf]

Supplementary Table 1. Baseline subgroup characteristics comparison according to the year of relapse.

Variable \ Value	Total n=1439	2003-2008 n=298	2009-2014 n=700	2015-2021 n=441	p-value
Age, years	62.7 (55.8-69.3)	62.0 (54.8-69.2)	62.3 (55.5-69.1)	63.5 (57.3-69.8)	0.09
Male, n (%)	865 (60.1)	183 (61.4)	418 (59.7)	264 (59.9)	0.88
Abnormal cytogenetics, n (%)	301/1226 (24.6)	71/240 (29.6)	152/634 (24.0)	78/352 (22.2)	0.10
High risk FISH, n (%)	249/1090 (22.8)	33/119 (27.7)	125/578 (21.6)	91/393 (23.2)	0.34
IgG isotype, n (%)	782 (54.3)	162 (54.4)	372 (53.1)	248 (56.2)	0.59
Involved kappa light chain, n (%)	844 (58.6)	172 (57.7)	399 (57.0)	273 (61.9)	0.24
Lytic lesions, n (%)	1013/1400 (72.4)	206/289 (71.3)	486/679 (71.6)	321/432 (74.3)	0.55
Plasmacytoma at diagnosis, n (%)	188/1397 (13.5)	36/287 (12.5)	76/676 (11.2)	76/434 (17.5)	**0.01**
Anemia, n (%)	454/1374 (33.0)	78/268 (29.1)	231/670 (34.5)	145/436 (33.3)	0.28
Hypercalcemia, n (%)	152/1278 (11.9)	24/237 (10.1)	77/624 (12.3)	51/417 (12.2)	0.65
Renal insufficiency, n (%)	207/1329 (15.6)	45/257 (17.5)	88/643 (13.7)	74/429 (17.3)	0.18
Serum albumin <3.5 g/dl, n (%)	541/1309 (41.3)	116/246 (47.2)	258/639 (40.4)	167/424 (39.4)	0.11
Serum beta-2-microglobulin, mg/l	4.1 (2.8-6.6)	4.0 (2.5-5.9)	3.9 (2.8-6.4)	4.4 (2.9-7.7)	**0.04**
Elevated lactate dehydrogenase, n (%)	182/960 (19.0)	26/183 (14.2)	85/459 (18.5)	71/318 (22.3)	0.08
ISS stage I, n (%)	338/1226 (27.6)	64/238 (26.9)	170/591 (28.8)	104/397 (26.2)	0.65
ISS stage II, n (%)	473/1226 (38.6)	106/238 (44.5)	222/591 (37.6)	145/397 (36.5)	0.10
ISS stage III, n (%)	415/1226 (33.8)	68/238 (28.6)	199/591 (33.7)	148/397 (37.3)	0.08

Supplementary Figure 1. First line treatment modalities

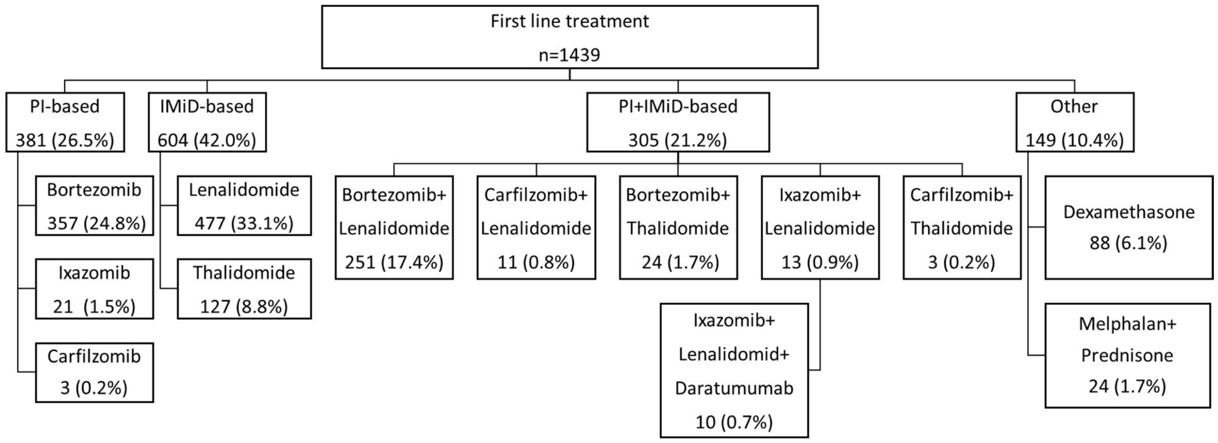

Supplementary Table 2. Detailed describtion of “other” 2nd line therapies, as depicted in *Figure 2.*

ACE (doxorubicin, cyclophosphamide, etoposide)	1
Benda-RD (bendamustine, lenalidomide, dexamethasone)	10
Bevacizumab	1
CD (cyclophosphamide, dexamethasone)	5
CDK Inhibitor	1
CHOP (cyclophosphamide, doxorubicin, vincristine, prednisone)	3
CP (cyclophosphamide, prednisone)	3
CRD (cyclophsophamide, lenaliodmide, dexamethasone)	13
CRVD (cyclophsophamide, lenaliodmide, bortezomib, dexamethasone)	1
CTD (cyclophsophamide, thalidomide, dexamethasone)	3
Cyclophosphamide monotherapy	4
C-VAMP (cyclophosphamide, vincristine, doxorubicin, methotrexate, prednisone)	1
CYBORD-P (cyclophosphamide, bortezomib, dexamethasone, pomalidomide)	1
CYBORD-R (cyclophosphamide, bortezomib, dexamethasone, lenalidomide)	2
Daratumumab+ ACE	1
Daratumumab-CD	1
Daratumumab-ICD (ixazomib, cyclophosphamide, dexamethasone)	4
Daratumumab-ID (ixazomib, dexamethasone)	1
Daratumumab-KD (carfilzomib, dexamethasone)	4
Daratumumab-KPD (carfilzomib, pomalidomide, dexamethasone)	1
Daratumumab-KRD (carfilzomib, lenalidomide, dexamethasone)	2
Daratumumab-VRD (bortezomib, lenalidomide, dexamethasone)	3
Dexamethasone monotherapy	17
D-PACE (dexamethasone, cisplatin, doxorubicin, cyclophosphamide, etoposide)	3
DT-PACE (dexamethasone, thalidomide, cisplatin, doxorubicin, cyclophosphamide, etoposide)	2
Elo-RD (elotuzumab, lenalidomide, dexamethasone)	7
Everolimus	1
HyperCVAD (cyclophosphamide, vincristine, doxorubicin, dexamethasone)	1
ICE (ifosfamide, cisplatin, etiposide)	1
ICD (ixazomib, cyclophosphamide, dexamethasone)	19
IPD (ixazomib, pomalidomide, dexamethasone)	10
KCD (carfilzomib, cyclophosphamide, dexamethasone)	4
KD (carfilzomib, dexamethasone)	6
KTD (carfilzomib, thalidomide, dexamethasone)	1
Melphalan-panobinostat	1
LCL161 with cyclophosphamide	1
Lenalidomide-cyclophosphamide-clarithromycin	1
MPR (melphalan, prednisone, lenalidomide)	4

MPT (melphalan, prednisone, thalidomide)	12
Nivolumab-daratumumab	1
Oprozomib-dexamethasone	1
Panobinostat-everolimus	1
Pembrolizumab-acalabrutinib	1
PVD (pomalidomide, bortezomib, dexamethasone	13
Solu-medrol	1
Sunitinib	3
TAD (thalidomide, doxorubicin and dexamethasone)	2
VAD (vincristine, doxorubicin, dexamethasone)	7
VBAP (vincristine, BCNU, doxorubicin, prednisone)	1
VBMCP (vincristine, carmustine, melphalan, cyclophosphamide, prednisone)	1
VDD (bortezomib, doxorubicin, dexamethasone)	12
VDD-thalidomide	1
Venetoclax-dexamethasone	2
VMP (bortezomib, melphalan, prednisone)	6
VMPT (bortezomib, melphalan, prednisone, thalidomide)	1
VPD (bortezomib, pomalidomde, dexamethasone)	5
Combined therapies:	
Daratumumab-dexamethasone//Daratumumab-RD (lenalidomide, dexamethasone)	1
Daratumumab-PD (pomalidomide, dexamethasone)//Daratumumab-KD	1
ID (ixazomib, dexamethasone)//VPD (bortezomib, pomalidomide, dexamethasone)	1
ERD (elotuzumab, lenalidomide, dexamethasone) //EPD (elotuzumab, pomalidomide, dexamethasone)	1
RD//CD	1
RD//CRD	1
RD/Elo-RD	1
RD//Melphalan-RD	1
TD (thalidomide, dexamethasone)//CTD	1
VD (bortezomib, dexamethasone)//VAD	1
VD//MP (melphalan, prednisone)	1
VTD (bortezomib, thalidomide, dexamethasone) //CYBORD //Daratumumab-RD	1
